# Anti-Cancer and Immunomodulatory Activity of a Polyethylene Glycol-Betulinic Acid Conjugate on Pancreatic Cancer Cells

**DOI:** 10.3390/life11060462

**Published:** 2021-05-21

**Authors:** Pascaline Nanga Fru, Ekene Emmanuel Nweke, Nompumelelo Mthimkhulu, Sindisiwe Mvango, Marietha Nel, Lynne Alison Pilcher, Mohammed Balogun

**Affiliations:** 1 Department of Surgery, School of Clinical Medicine, Faculty of Health Sciences, University of the Witwatersrand, Johannesburg 2193, South Africa; ekene.eweke@wits.ac.za (E.E.N.); mthimkhuluportia@gmail.com (N.M.); marietha.nel@wits.ac.za (M.N.); 2 Biopolymer Modification and Therapeutics Laboratory, Chemicals Cluster, Council for Scientific and Industrial Research, Meiring Naude Road, Brummeria, Pretoria 0001, South Africa; sindisiwe.sindisiwe.mvango@gmail.com (S.M.); mbalogun@csir.co.za (M.B.); 3 Department of Chemistry, University of Pretoria, Pretoria 0002, South Africa; lynne.pilcher@up.ac.za

**Keywords:** betulinic acid, polyethylene glycol, apoptosis, pancreatic cancer, polymer therapeutics

## Abstract

Drug delivery systems involving polymer therapeutics enhance drug potency by improved solubility and specificity and may assist in circumventing chemoresistance in pancreatic cancer (PC). We compared the effectiveness of the naturally occurring drug, betulinic acid (BA), alone and in a polymer conjugate construct of polyethylene glycol (PEG), (PEG–BA), on PC cells (MIA PaCa-2), a normal cell line (Vero) and on peripheral blood mononuclear cells (PBMCs). PEG–BA, was tested for its effect on cell death, immunomodulation and chemoresistance-linked signalling pathways. The conjugate was significantly more toxic to PC cells (*p* < 0.001, IC_50_ of 1.35 ± 0.11 µM) compared to BA (IC_50_ of 12.70 ± 0.34 µM), with a selectivity index (SI) of 7.28 compared to 1.4 in Vero cells. Cytotoxicity was confirmed by increased apoptotic cell death. PEG–BA inhibited the production of IL-6 by 4–5.5 fold compared to BA-treated cells. Furthermore, PEG–BA treatment of MIA PaCa-2 cells resulted in the dysregulation of crucial chemoresistance genes such as *WNT3A*, *TXNRD1*, *SLC2A1* and *GATA3*. The dysregulation of chemoresistance-associated genes and the inhibition of cytokines such as IL-6 by the model polymer construct, PEG–BA, holds promise for further exploration in PC treatment.

## 1. Introduction

Pancreatic cancer (PC) is one of the world’s deadliest cancers, with an increasing incidence and mortality rate. In 2018, PC was the third-leading cause of cancer-related deaths in the United States [1] and the seventh in other high-income countries [2]. There is, however, very little difference in the survival rates among countries, irrespective of income category, with an average survival rate of 5% over five years [3]. Forecasts suggest that by 2030 PC will be the second-leading cause in the US [4], while in the European Union it will surpass breast cancer to become the third-leading cause of cancer-related deaths by 2025 [5].

To manage PC, gemcitabine and FOLFIRINOX are currently used individually or in combination therapies in two separate regimens with drugs such as nab-paclitaxel and 5-fluorouracil (5-FU), or with radiotherapy [6,7,8,9]. Unfortunately, these two regimens only result in a marginal life expectancy extension of about 6-8 months and are associated with increased toxicity [8]. Gemcitabine is known to induce nephrotoxicity and systemic toxicity [10]. Consequently, despite efforts to develop improved chemotherapeutic strategies for PC, the overall survival rate has not improved in the last five decades. This persistent high mortality and the toxic effects of existing drugs suggest a need for new chemotherapeutic agents with better therapeutic indices.

Betulinic acid (BA) is a naturally occurring pentacyclic triterpene found in the bark of the *Betula alba* (white birch) tree [11]. Its selective inhibition of melanoma cells was first reported in the 1990s [12]. BA is known to exhibit potent anti-cancer properties with selective cytotoxicity by inducing apoptosis via various pathways including nuclear factor-kappa beta (NF-_K_B), MAPK and mitochondrial pathways [13]. The hallmark of most human cancers, including PC, is the upregulation of NF-_K_B, which has been shown to play a role in causing resistance to chemotherapy and radiotherapy [14]. BA suppresses carcinogenic NF-_K_B activation by inhibiting IkB [15] and triggering permeabilisation of the mitochondrial membrane to release proteins that activate caspase 3, thus leading to the activation of apoptosis [16,17,18]. A crucial advantage of BA and its analogues is that their mechanisms of action involves the direct killing of cells, circumventing biological pathways or processes that often raise chemoresistance to other drugs [13].

The apoptotic inducing activity of BA as an anti-cancer agent is enhanced by the ability to inhibit cell growth and metastasis in various cancer cells, including PC, selectively [11,19,20,21,22,23,24] and to reverse nephrotoxicity [25]. However, BA has the disadvantages of poor aqueous solubility [26] and reduced plasma half-life, the Achilles heel of many potential drug candidates [27]. In one attempt to improve the solubility of BA, Jeong and colleagues conjugated the molecule to several amino acids [28]. The result was a variation in the improvement of aqueous solubility and cytotoxicity of BA.

Nanomedicine delivery systems have also been explored, as with other drugs, to improve the solubility and pharmacological performance of BA simultaneously [29]. Various delivery system architectures and formulations, including polymeric nanoparticles, magnetic nanoparticles, liposomes, emulsions, cyclodextrin complexes, and polymer–drug conjugates have been reported [30]. In particular, polymer–drug conjugates are of interest because a hydrophilic polymer such as polyethylene glycol (PEG) creates a new water-soluble chemical entity [31]. In contrast, liposomes and nanoemulsions are thermodynamically stable biphasic aqueous solutions of drug-containing solids. Often, the conjugation of a bioactive drug molecule to a polymeric carrier results in an inactive prodrug. Activity is restored on the release of the drug. Dai and colleagues conjugated BA to an 8-arm PEG with a 290–750 fold increase in aqueous solubility [32]. The authors reported that the prodrug demonstrated “excellent in vitro anti-cancer activity”. The ester linkage between the polymer and the BA would imply that the free drug is released intracellularly by pH-dependent hydrolysis after uptake by pinocytosis [32]. Saneja and colleagues also reported on a pegylated BA conjugate [27]. We recently reported the synthesis and physicochemical characterisation of a linear PEG–BA conjugate linked via an amide bond [33], which is less readily released by pH-dependent hydrolysis [33], such as that occurring in the extracellular space. Using amide bonds in the conjugation of PEG–BA, therefore, affords improved intracellular delivery of the BA moiety in addition to the aforementioned advantages of such a system. Other previously reported cytotoxicity studies used a biodegradable ester-conjugated PEG–BA system to achieve similar results [27,32]. PEGylation with other natural-based compounds such as oleanolic and maslinic acid resulted in improved solubility and activity of the parent compound [34].

In this study, we investigate the PEG–BA prodrug as a polymeric construct to provide insight into intracellular interactions and effects of the entire conjugate system relative to free BA. The study is further supported by the fact that currently published articles have not reported on the intracellular molecular targets specifically concerning the robust interrogation of chemoresistance and associated signalling pathways of polymer-BA conjugates, especially in PC cells. Inhibiting or minimising potential chemoresistance is crucial, not just for BA but for any chemotherapeutic agent. Therapeutic targets to prevent chemoresistance for proteins such as cytokines and genes are instrumental in this quest. IL-6, a cytokine found in high concentrations in the tumour microenvironment, has been implicated in causing chemoresistance in cancers [35,36]. Similarly, there are genes such as those of the *WNT3A* and oxidative stress pathways that are reportedly important components for the progression and chemoresistance seen in cancers [37,38].

Given the continued increasing incidence and problems of managing PC, the purpose of this study was to compare the effectiveness of the PEG–BA conjugate over the free drug BA on the activity of PC as a potential avenue for treatment. We made use of a PC cell line (MIA PaCa-2), a normal cell line (Vero cells) and primary cells (PBMCs). Increased cell death and improved apoptosis and dysregulation of vital genes involved in chemoresistance and downregulation of the proinflammatory cytokine IL-6 were notable for the drug conjugate compared to the free drug against these cells.

## 2. Materials and Methods

### 2.1. Reagents

Materials for this study were of analytical grade, and the sources are specified on first mention.

### 2.2. Polymer Conjugation and Compound Preparation

BA was covalently linked via an amide bond to PEG as we previously reported [33]. 1-Ethyl-3-(3-dimethylaminopropyl)carbodiimide was used as the carboxyl activating agent in a one-pot reaction for 24 h.

The compounds, BA and PEG–BA, were dissolved in DMSO (Sigma Aldrich, St Louis, Missouri, USA) to a concentration of 20 mg/mL, and stored in single-use aliquots at −20 °C until needed for biological assays. The compounds were further diluted with complete Dulbecco’s Modified Eagle Medium (DMEM) from Sigma Aldrich (St. Louis, MO, USA) to a concentration of 1 mg/mL. Experimental concentrations ranged from 0.4 to 100 µM. The final concentrations of DMSO (<0.5%) in the treatments had minimal cytotoxicity and control wells treated with DMSO only, were used as vehicle controls (untreated cells).

### 2.3. Cell Culture

MIA PaCa-2 [39] (a pancreatic cancer cell line) and Vero cells [40] (a non-tumorigenic primary monkey kidney cell line commonly used as a control cell line for cytotoxicity assays in drug studies) were used in this study. The MIA PaCa-2 cells were obtained from the Japanese National Institutes of Biomedical Innovation Cell Bank and Vero cells from Highveld Biological, Johannesburg, South Africa. The cells were grown in 75 cm^2^ culture flasks (BD Biosciences, San Diego, CA, USA) and maintained (37 °C, 5% CO_2_, 95% humidity) in complete DMEM supplemented with 100U antibiotic/antimycotic solution and 10% heat-inactivated (56 °C for 30 min) foetal bovine serum (FBS), all from Sigma Aldrich. The cells were harvested for use in bioassays at 90–100% confluency.

### 2.4. Cytotoxicity Assay

The cytotoxic effect of BA and PEG–BA was assessed using the 2,3-bis-(2-methoxy-4-nitro-5-sulfophenyl)-2H-tetrazolium-5-carboxanilide (XTT) assay, an improved tetrazolium dye based assay that obviates the solubilisation step [41]. A concentration of 1 × 10^5^ cells/mL each of adherent MIA PaCa-2 and Vero cells were seeded into 96-well plates (BD Biosciences, San Diego, California, USA) overnight before treatment with the compounds at concentrations ranging from 0.4 to 100 µM for 72 h. Doxorubicin (0.2 µg/mL) was used as a positive control for cell death assessment and DMSO was used as a vehicle control. After treatment, washed cells (433× *g*, 5 min, 25 °C) were exposed to 125 µL of complete DMEM containing 25 µL of XTT solution and incubated (37 °C, 5% CO_2_, 95% humidity) for 4 h. The optical density of the mixture was measured at 450 nm with a background at 690 nm using a Multiskan Ascent 96/384 microplate reader (Labsystem, Vantaa, Finland). Cell viability was determined as a percentage relative to the vehicle control. The IC_50_ was then calculated using GraphPad Prism 6 (GraphPad Software, Inc, San Diego, CA, USA). The selectivity index (IC_50_ Vero cells/IC_50_ MIA PaCa-2) and potentiation factor (PF_50_) at 50% growth inhibition (IC_50_) were also determined. The PF_50_ is the IC_50_ value of free drug divided by the IC_50_ value obtained from a combination of drugs [42], which is the polymer drug conjugate in this case. Statistical significance was calculated using a two-way analysis of variant (ANOVA) and the Bonferroni post-test to compare replicate means in GraphPad Prism 6 (GraphPad Software, Inc, San Diego, CA, USA). A *p* < 0.05 was considered to be statistically significant.

### 2.5. Apoptosis Detection Using Annexin V Apoptosis Detection Kit

The mode of cell death induced by the BA and PEG–BA on MIA PaCa-2 and Vero cells was assessed using the Annexin V-FITC Apoptosis kit I (BD Biosciences, San Diego, CA, USA). The assay was performed per the manufacturer’s protocol [43] and as previously described [44] with slight modifications. The cells were analysed for apoptosis and necrosis after acquiring 30,000 events using a BD LSRFortessa™ Analyser (BD Biosciences, San Diego, CA, USA). The BD FACSDiva software was used to detect fluorescein isothiocyanate (FITC) positive signals for apoptotic cells (Annexin-v positive) on a 530/30 filter and peridinin–chlorophyll–protein (PerCP) positive signals for necrotic cells (propidium iodide positive) on a 610/20 band pass filter. Apoptosis was determined at three different concentrations around the IC_50,_ 1, 1.7 and 4 µM. Doxorubicin (0.2 ug/mL) was used as a positive control for apoptosis induction. The data were exported as flow cytometry standard (FCS) files and analysed using FlowJo software Version 10 (FlowJo, LLC, Ashland, OR, USA). The data were exported and represented as means ± standard error of three independent experiments using GraphPad Prism 6.

### 2.6. Cytometric Bead Array Kit for Measuring Th1Th2Th17 Cytokine Frequency

The supernatant obtained from PBMCs (1 × 10^6^ cells/mL) treated and incubated (72 h, 37 °C, 5% CO_2_, 95% humidity) with BA and PEG–BA (3 µM) were used for this assay. Cytotoxicity was performed on isolated PBMCs (see Supplementary Materials for isolation protocol) using the 3-(4,5-dimethylthiazol-2-yl)-2,5-diphenyl tetrazolium bromide (MTT) assay as previously described [45]. For the cytometric bead array (CBA) assay, the PBMCs were isolated from both consenting PC patients (*n* = 4) and healthy control donors (*n* = 2). Ethics clearance to collect blood samples was obtained from the Human Research Ethics Committee (Medical) of the University of the Witwatersrand (M170440). The PBMCs were stimulated with 2 µg/mL of phytohaemagglutinin-protein (PHA-P). After the 72 h incubation period, the cells and medium were transferred to 5 mL Falcon^®^ round-bottom polypropylene tubes and centrifuged at 433× *g* for 5 min at room temperature. Aliquots of the cell-free supernatant were collected and stored at −80 °C until needed for experiments.

The CBA Th1/Th2/Th17 assay was performed according to the manufacturer’s instructions (BD Biosciences, La Jolla, San Diego, CA, USA) and as previously described [46], but with minor modifications. In summary, unique antibody-coated beads were used to bind to cytokines present in the cell-free supernatant. Specific fluorescence associated with each bead population was detected using flow cytometry allowing for the identification of the various cytokines in the sample. A standard assay-specific template provided by BD Biosciences was used to acquire 10,000 events per sample on an LSRFortessa™flow cytometer (BD Biosciences, San Diego, CA, USA). Acquired FCS files were analysed using a flow cytometric analysis software (FCAPArray™) from BD Biosciences. Interferon-gamma (IFN-γ), TNF-α, IL-2, IL-4, IL-6, IL-10 and IL-17A concentrations were all determined from standard curves. Our focus here was primarily on the proinflammatory cytokine, IL-6.

### 2.7. Total RNA Extraction

MIA PaCa-2 and Vero cells were treated with 1.7 µM BA and PEG–BA. The cells were harvested, and 1 mL of TRI Reagent^®^ (Sigma Aldrich St. Louis, MI, USA) was used to lyse them [47]. Samples were allowed to rest for 10 min at room temperature followed by the addition of 200 μL of chloroform (Sigma Aldrich, St. Louis, MI, USA). The mixture was shaken thoroughly for 15 s and allowed to rest at room temperature for 15 min before centrifugation at 12,000× *g* for 15 min at 4 °C. The aqueous supernatant was carefully transferred into a fresh 1.5 mL tube, and 500 μL of isopropanol (Sigma Aldrich, St. Louis, MI, United States) added and left to rest at room temperature for 10 min. The sample was centrifuged at 12,000× *g* for 10 min at 4 °C to pellet the RNA, which was washed with 1 mL 75% ethanol (Sigma Aldrich, St. Louis, MI, United States) at 7500× *g* for 5 min at 4 °C. The pellet was left to air-dry at room temperature for 10 min and was then dissolved in nuclease-free water (Qiagen, Hilden, Germany). Total RNA quantity and quality was determined using the Nanodrop 2000 instrument (ThermoFischer Scientific, Waltham, MA, USA).

### 2.8. Genomic DNA Elimination and Complementary DNA (cDNA) Synthesis

The genomic DNA elimination and cDNA synthesis steps were conducted using the RT^2^ First Strand cDNA synthesis kit (Qiagen, Hilden, Germany). The genomic DNA elimination mix was prepared from 2 μg of the total RNA, 2 μL of genomic DNA elimination buffer (Buffer GE) and nuclease free water up to a mixture total final volume of 10 μL. The mixture was incubated for 5 min at 42 °C and immediately placed on ice. The cDNA synthesis was performed according to the manufacturer’s instructions outlined in the protocol.

### 2.9. Differential Gene Expression and Statistical Analyses

The Human Signal Transduction PathwayFinder RT^2^ Profiler PCR array (Qiagen, Hilden, Germany) was used to identify chemoresistance-linked pathways affected by treatment with BA and PEG–BA. This array contains crucial genes responsible for the activation or inhibition of several signalling processes involved in development, metabolism, immunology and stress-stimulation. It comprises five reference genes, one genomic DNA contamination control, three reverse transcription controls and three positive PCR controls. The sample mixture was prepared, and the assay performed according to the manufacturer’s instruction. A real-time PCR was conducted using the Bio-Rad CFX96 real-time touch detection system (Bio-Rad, Hercules, CA, USA). The CFX Maestro™ was used to generate Ct values and the Qiagen RT^2^ PCR data analysis portal (https://geneglobe.qiagen.com/za/analyze/ (accessed on 12 May 2021)) used for differential gene expression analysis. The Qiagen tool was used to identify differentially expressed genes obtained by comparing PEG–BA-treated to BA-treated cells. This tool calculated fold change using the delta delta CT (2^−ΔΔCT^) method [48].

## 3. Results

### 3.1. PEG–BA Results

**Figure 1:**
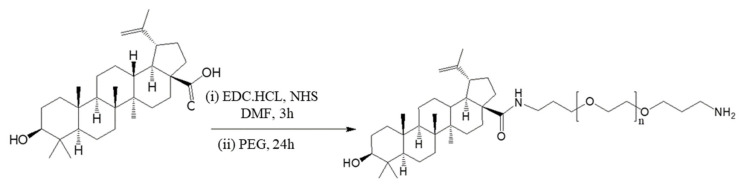
Schematic of PEG–BA synthesis.

Details of the synthesis and characterisation were previously reported by us (Mvango et al., 2020) [33]. Figure 1 shows the structures and processes involved in the synthesis of BA and PEG–BA. There was a 64% recovery yield of the PEG–BA after purification. Confirmation of the PEG–BA conjugation was done by ^1^H NMR spectroscopy where the terpenoid protons of BA and the –O–CH_2_–CH_2_– and the methylene protons of CH_2_–NH_2_ of PEG were successfully assigned.

### 3.2. PEG–BA Induces Increased Dose-Dependent Cytotoxicity in MIA PaCa-2 Cells

**Figure 2:**
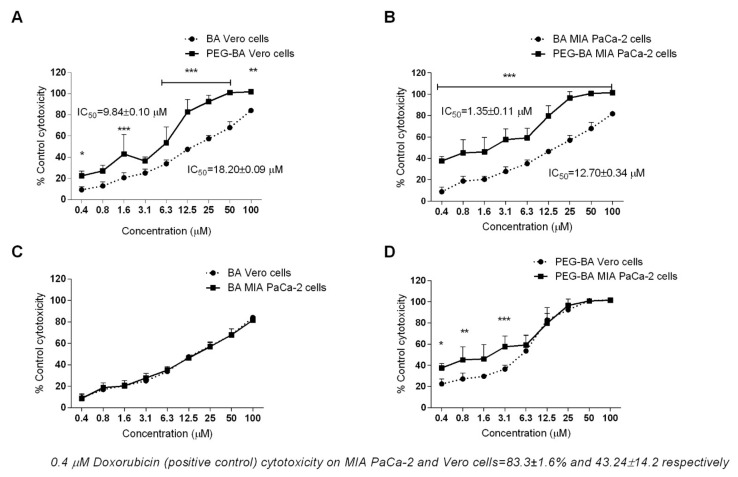
The effects of BA, PEG–BA on the viability of Vero cells (**A**) and MIA PaCa-2 cells (**B**). Cells were treated with compounds (0.4-100 µM) for 72 h and 2,3-bis-(2-methoxy-4-nitro-5-sulfophenyl)-2H-tetrazolium-5-carboxanilide (XTT) added for a further 4 h to detect viable cells. When compared to BA, PEG–BA caused a significant decrease in Vero cell viability (**A**) at selected concentrations (0.4, 1.6, 6.25–100 µM) and over the entire concentration range from 0.4–100 µM (p < 0.003) in the MIA PaCa-2 cells. The conjugate inhibited the growth of MIA PaCa-2 cells more than Vero cells with BA being less cytotoxic (**C**) compared to PEG BA (**D**). At the lowest concentration (0.4 µM), the positive control, doxorubicin was more toxic than BA and BA-PEG and more specific for to MIA PaCa-2 cells (83.3 ± 1.6%) than Vero cells (43.24 ± 14.2). The results are from five independent experiments performed in duplicate. Statistical significance was determined using a two-way analysis of variance (ANOVA) and the Bonferroni post-test to compare replicate means. * *p* = 0.043, ** *p* = 0.004, *** *p* < 0.0001.

The cytotoxic effect of BA and PEG–BA was determined by studying the viability of MIA PaCa-2 and Vero cells using XTT. A dose-dependent cytotoxic effect relative to the untreated cells, was observed (Figure 2). Unlike in Vero cells (Figure 2A), PEG–BA was significantly more cytotoxic than BA to MIA PaCa-2 cells (*p* < 0.001) at all tested concentrations with an IC_50_ of 1.35 ± 0.11 µM compared to 12.70 ± 0.34 µM, respectively (Figure 2B). Although PEG–BA was significantly more toxic to Vero cells than BA at most of the tested concentrations, the 50% inhibitory concentration was higher than for MIA PaCa-2 cells (IC_50_ of 9.84 ± 0.10 µM and 18.20 ± 0.09 µM, respectively, Figure 2B). These findings suggest that the conjugate, PEG–BA, was cytotoxic to 50% of MIA PaCa-2 cells at a concentration 7.3 times lower than that required to kill 50% of the Vero cells. This value is also known as the selectivity index (SI). The SI index for BA on the other hand was 1.43. When the individual compounds were tested in both cell lines, BA (Figure 2C) showed little to no selectivity to either MIA PaCa-2 cells or Vero cells compared to PEG–BA (Figure 2D) which was selectively more toxic to MIA PaCa-2 cells (*p* < 0.05 at 3.125 µM and lower). Furthermore, the IC_50_ of BA was higher than that of PEG–BA in MIA PaCa-2 cells and in Vero cells, suggesting an increased efficacy of PEG–BA with a PF_50_ of 9.4 and 1.85 respectively. Doxorubicin was much more toxic to MIA PaCa-2 cells than to Vero cells inducing cell death by more than 80% (83.3 ± 1.6%) compared to 43.24 ± 14.2 at 0.2 µg/mL (0.4 µM), a concentration far lower than that seen for PEG–BA. However, with, doxorubicin’s inherent cardiotoxic side effects, the PEG-BA polymer conjugate should be a better drug [49]. There were no significant differences in the effect of BA on the two different cell types (Figure 2C).

### 3.3. PEG–BA Induces Apoptosis in MIA PaCa-2 Cells

**Figure 3:**
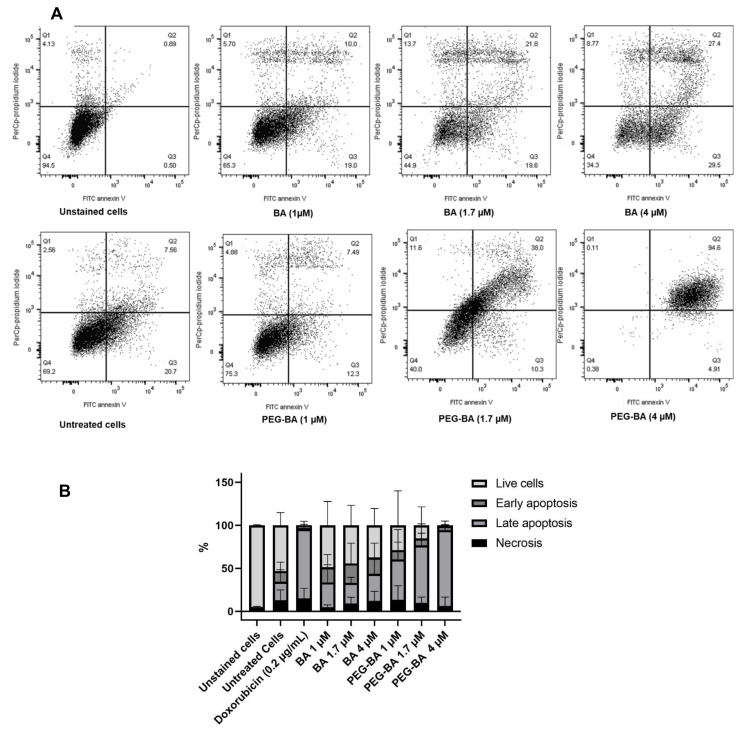
Apoptotic and necrotic effect of BA and PEG–BA on MIA PaCa-2 cells. Representative dot plots showing apoptosis at varying concentrations of BA and PEG–BA are shown in A. The cells were treated with the compounds for 72 h, and cell death measured using Annexin V and propidium iodide staining by flow cytometry (**A**). Fluorescein isothiocyanate (FITC) channel detected annexin-V+ stained apoptotic cells (*x*-axis) while peridinin–chlorophyll–Protein (PerCP) channel detected propidium iodide or necrotic cells (*y*-axis). Apoptotic cells represented in Q2 and Q3 as late and early apoptosis were separated from necrotic cells in Q1 and live cells in Q4. (**B**) A quantitative stacked bar graph showing the apoptosis induced by BA and PEG–BA on MIA PaCa-2 cells. PEG–BA caused a higher percentage of apoptosis (mainly shown as late apoptosis) compared to free BA. The cells of interest were gated from singlet cells and debris using forward and side scatter properties. Untreated cells represent the vehicle control (DMSO), *n* = 3.

Apoptosis induction was determined using double staining with Annexin V conjugated to FITC, and PI. Both compounds showed dose-dependent apoptotic effects on MIA PaCa-2 cell. Early and late apoptosis ranged on average between 17 and 22% and 24 and 31% for BA-treated cells and 5–9% and 46–88% for PEG–BA-treated cells, respectively (Figure 3). In PEG–BA-treated cells, a higher percentage of cells were in late apoptosis, suggesting higher toxic effects to the cells than from BA, as shown in representative dot plots (Figure 3A) and the quantitative findings (Figure 3B). These findings further confirm the observed results from the cytotoxicity studies (Figure 2), where similar concentrations of BA resulted in lower toxicity compared to PEG–BA, hence the differences in IC_50_. At a concentration of 4 µM both BA (31.43 ± 16.7%) and PEG–BA (88.03 ± 6.5%) showed the highest apoptotic effect (although mainly in the late-apoptotic phase) on MIA PaCa-2 cells (Figure 3). The cell death profile observed for PEG–BA at 4 µM was similar to that seen for the positive control, doxorubicin, at a 10 times lower concentration (0.4 µM).

### 3.4. PEG–BA Inhibits IL-6 Production from PBMCs

**Figure 4:**
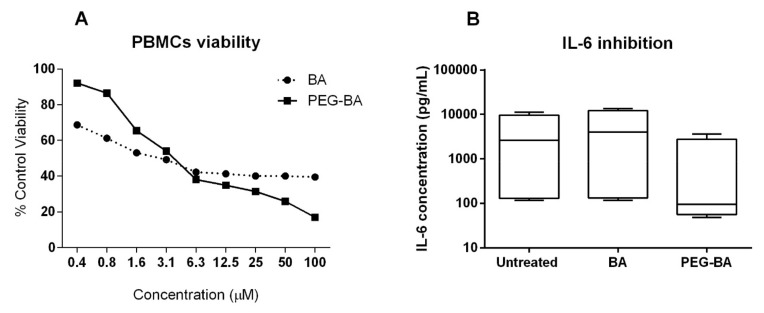
Effect of BA and PEG–BA on IL-6 produced by peripheral blood mononuclear cells (PBMCs). The effect of BA and PEG–BA on PBMC viability is shown in (**A**). A dose-dependent effect is observed. At 3 µM, PEG–BA inhibited IL-6 by 4 and 5.5 fold more compared to untreated cells and BA-treated cells (**B**), respectively. This was the case despite both compounds having closely similar effects on PBMC viability (53.9 ± 0.7% and 9.2 ± 0.8% respectively), *n* = 4. Data are plotted to a scale of log10 on the *y*-axis.

Similar cytotoxicity profiles were observed when PBMCs were treated with a 3 µM concentration of BA (49.2 ± 0.8%) and PEG–BA (54 ± 0.7%), respectively, as shown in Figure 4A. Despite the very similar cytotoxic effects on these cells, there was a consistent decrease in IL-6 production from cells of both control and patient samples treated with PEG–BA (Figure 4B). Also notable was the fact that, despite the heterogeneity between individual patient samples (*n* = 4), potentially stemming from differences in cancer severity between the patients (locally advanced versus metastatic), the inhibitory effect induced by PEG–BA was consistent for both the control and patient samples (Figure S1). On average, there was a four-fold decrease in IL-6 from PEG–BA-treated cells compared to BA-treated cells (971.15 pg/mL compared to 5430.94 pg/mL). Decreases in IL-6 production were also observed when cells from control participant samples (*n* = 2) were treated with PEG–BA and BA (10.59 pg/mL compared to 396.24 pg/mL), respectively. For most of the other tested cytokines, inhibition by PEG–BA was higher than that caused by BA (Figure S2). Overall, the expression level of all seven cytokines from all patient samples was higher compared to the control samples, probably due to the higher inflammatory response in these patients (Figure S2).

### 3.5. PEG–BA Treatment Dysregulates Key Genes Involved in Chemoresistance

**Figure 5:**
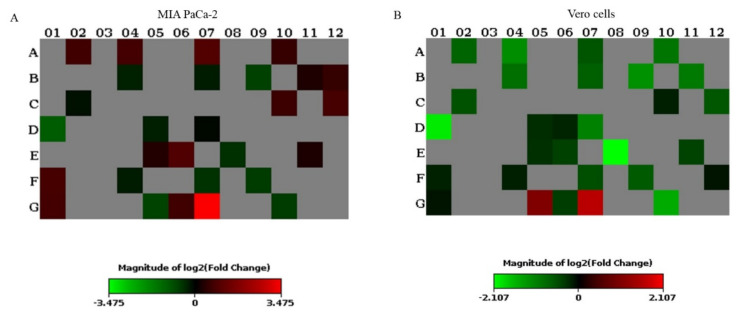
Gene expression profiling of PEG–BA-treated, and BA-treated Vero and MIA Paca-2 cells. Heat map showing gene expression in (**A**) MIA PaCa-2 (**B**) Vero PEG–BA compared to BA-only treated cells. The cut-off fold change was set at 1.5. The range of the magnitude of fold changes are colour-coded from green to red. Grey colour coding indicates genes with no changes in expression. The plate layout showing the position of all genes can be seen in Figure S3.

Vero and MIA PaCa-2 cells treated with PEG–BA were compared to BA-treated cells and the expression profiles of several genes in diverse signalling pathways were determined (Figure 5) to gain a better understanding of the action mechanism of the drugs. MIA PaCa-2 cells treated with PEG–BA upregulated 11 genes involved mainly in hypoxia, oxidative stress and WNT signalling, and downregulated five genes (*GATA3*, *TXNRD1*, *CDKN1A*, *WNT3A* and *SLC2A1*). In PEG–BA-treated Vero cells, we observed the upregulation of three genes (*WISP1*, *ACTB* and *TXNRD1*) and the downregulation of 15 genes (involved in pathways such as JAK/STAT, p53 and hedgehog signalling) (Table 1). Genes such as *WISP1* and *ACTB* were commonly upregulated in both the Vero and MIA PaCa-2 cells treated with PEG–BA, and *GATA3* and *CDKN1A* were commonly downregulated. Furthermore, while *TXNRD1* was upregulated in PEG–BA-treated Vero cells, it was downregulated in MIA PaCa-2 cells. In contrast, *AXIN2* and *GADD45B* were downregulated in Vero cells but upregulated in MIA PaCa-2 cells.

## 4. Discussion

The findings from this study demonstrated that compared to free BA, PEG–BA is more cytotoxic, anti-proliferative and induces apoptosis in PC cells. A lower IC_50_ and a significantly more cytotoxic effect was observed when these cells were treated with PEG–BA, suggesting increased conjugate potency (Figure 2). The lower IC_50_ correlated with increased apoptotic phosphatidylserine exposure (Figure 3). Although this study confirmed that BA was toxic to MIA PaCa-2 cells and led to apoptosis, a higher BA concentration was required to kill 50% of these PC cells, suggesting it had less potency than PEG–BA. Notably, both BA and PEG–BA were less toxic to the normal cells (Figure 2A,B), indicating increased specificity to cancer cells. Similar to the current findings, the inhibition of pancreatic cancer by BA was recently reported by Sun and colleagues in a 2019 paper, where they showed that BA inhibited the stem-like phenotype in PC cells [22]. However, the improved anti-cancer activity of BA by conjugation with PEG is a noteworthy improvement in this area of study. Other reports of BA activity on cancers such as human liver cancer cell lines (HUH7 and PLC/PRF/5) showed that BA induced apoptosis by targeting the p53 signalling pathway [50]. In other studies, confirmation of the apoptosis-inducing ability of BA in murine melanoma cells by activating the intrinsic mitochondrial pathway and modulating the NF-κB were reported [13,51].

In vitro cytotoxicity to normal cells potentially means in vivo system toxicity, resulting in adverse side-effects, an unfortunate limitation of conventional chemotherapeutic treatment. Interestingly, just over seven times more of the conjugate, PEG–BA, was needed to kill normal cells (1.35 ± 0.11 µM to 9.84 ± 0.10 µM) whereas just under two times of the free drug was required to kill normal cells compared to the PC cells (12.70 ± 0.34 µM to 18.20 ± 0.09 µM) (Figure 2). A notable finding was that the dose-dependent cytotoxicity profile of BA and PEG–BA look very similar (Figure 2). This could be attributed to the fact that PEG mainly plays a delivery role, with the active drug being BA in both instances.

These findings reiterate the potential of conjugation for improving specificity to PC cells. Drug–polymer conjugation has the advantage of transporting the drug in its inactive form until it reaches the target site [52]. The polymer helps the drug to escape the monocyte phagocytic system. It enables the delivery of adequate amounts of the drug to the target site, reducing non-specificity and associated side-effects [53]. In the case of the PEG–BA conjugate here, the amide bond used in the conjugation resulted in improved delivery of free BA into the intracellular space.

PEG–BA potentiates apoptosis as seen by an increase in phosphatidylserine exposure on MIA PaCa-2 cells (Figure 3). Initial results suggest that this could be associated with an increase in caspase 3/7 activity (data not shown). The findings suggest the increased ability of the polymer conjugate drug to permeate mitochondria and cause the release of cytochrome c, which triggers apoptotic protease activating factor 1, leading to the formation of apoptosomes [54,55]. It is well established that PEGylation results in improved solubility, which assists in carrying hydrophobic drugs by enhancing aqueous solubility [56,57,58]. Having shown that PEG–BA is more toxic to cancer cells than normal cells, future work will include cellular uptake studies to illustrate the uptake of conjugated BA compared to free BA.

Chemotherapeutic resistance is characteristic of PC as with many cancers. Unlike BA, which notably exhibited a similar cytotoxicity profile on PBMCs, when these cells were treated with PEG–BA, there was consistent inhibition of IL-6 expression, irrespective of cell source (control donors or patient samples). This finding suggests that the effect of PEG–BA on IL-6 was not because of its cytotoxic effect but rather improved activity because of conjugation. It is also important to note that toxicity on PBMCs exhibited similar profiles probably because these were normal cells, a response also seen for Vero cells (Figure 2C). Given the resultant dysregulation and abundance of IL-6 in the tumour microenvironment, the inhibition of IL-6 as a therapeutic target in cancer progression may be beneficial [59].

Our results also show that distinct genes were dysregulated when PEG–BA-treated Vero and MIA PaCa-2 cells were compared to BA-treated cells, respectively. Importantly, treatment with PEG–BA led to the dysregulation of key genes associated with chemotherapeutic resistance and PC progression (Figure 5, Table 1). *WNT3A*, shown to be downregulated by PEG–BA in MIA PaCa-2 cells, is an initiator of the WNT pathway, implicated in PC chemoresistance [60,61,62]. Interestingly, the study showed the simultaneous upregulation of *Axin*, an important modulator of the WNT pathway, in PEG–BA-treated PC cells. Downregulation or degradation of *Axin* increases the activity of the WNT pathway [63]. *TXNRD1*, was downregulated in PEG–BA-treated PC cells but upregulated in normal cells, where it protects against oxidative stress and is required for tissue growth and development; therefore, its overexpression allows for continued normal cell growth. *TXNRD1* was highly expressed in chemoresistant PC cells, and its inhibition enabled chemosensitivity [64]. The dysregulation of these genes indicated the potential role of PEG–BA to enhance chemosensitivity in PC cells.

The study further showed that genes such as *SLC2A1* and *GATA3* were downregulated in PC cells treated with PEG–BA. These genes are overexpressed and associated with poor survival in PDAC patients [65,66]. *SLC2A1* (Glut1) is an essential cellular glucose transporter providing a crucial constant and quick source of energy, enhancing cellular proliferation. A hallmark of cancer is increased glucose uptake and several studies have linked this to chemoresistance [67,68]. In this study, PEG–BA downregulated *SLC2A1* and may decrease cellular glucose uptake effectively, depriving cancer cells of much-needed energy to grow and proliferate.

Both *FTH1* and *STAT1* were upregulated in PEG–BA-treated MIA PaCa-2 cells. *FTH1* is responsible for reducing iron in cells. Cancerous cells require large amounts of iron to continue proliferating [69]; a reduction in *FTH1* would inhibit cell proliferation. Additionally, the loss of *STAT1* has been linked to poor cancer prognosis and metastasis [70]; therefore, we hypothesise that upregulating *STAT1* may have a contrary effect.

In PC, bypassing the stroma is a significant limitation to treatment. Polymer–drug conjugates offer an opportunity to bypass the stroma since they enable the addition of moieties that can degrade and thus bypass the dense structure. The targeting moiety may be an agent, which can function similarly to gemcitabine, taken up via the human equilibrative nucleoside transporter 1 (hENT1). The desmoplastic stroma also comprise an extracellular matrix rich in glycosaminoglycans such as hyaluronic acid (HA), contributing to treatment resistance in PC [71]. Polymer therapeutics using PEGylated hyaluronidase, which targets and degrades HA within the tumour microenvironment in pancreatic adenocarcinomas, has shown promising results [71]. However, a reported disadvantage of PEG and potentially PEG in PEG–BA, is that it is non-biodegradable [72], and this property should be considered when conjugating. However, reports have suggested that PEG undergoes oxidative degradation under appropriate biological conditions, which may contribute to how conjugates of PEG influence processes in the cell [72]. While the data reported here is a first step towards developing a PEG construct with improved anti-cancer activity in our lab, the likelihood of PEG–BA biodegradation under appropriate biological conditions is possible and encouraging, according to Ulbricht and colleagues [72].

A possible limitation with this study is that Vero cells, a non-tumorigenic primary monkey kidney cell line, was used as a control cell line instead of a normal human cell line. Given the similar responses observed for both BA and PEG–BA on this cell line (Figure 2C) and to PBMCs (Figure 4A), it seems justifiable to consider them as normal cells in this study.

## 5. Conclusions

Over and above showing the anti-cancer activity of BA on its own, this proof-of-concept study also showed improved potency and specificity of PEG–BA on the MIA PaCa-2 PC cell line compared to BA. Our data confirmed the potential of polymer conjugation for increasing the PC-inhibitory activity of BA by enhancing apoptosis, reducing cellular proliferation and possibly circumventing chemoresistance. This study encourages the development of more potent and specific naturally occurring anti-cancer drugs for clinical PC treatment.

## Figures and Tables

**Table 1:** Dysregulated genes in each pathway represented in the array and their fold changes in PEG–BA-treated compared to BA-treated Vero and MIA PaCa-2 cells.

Pathways	Upregulated (PEG–BA vs. BA)				Downregulated (PEG–BA vs. BA)			
	Vero		MIA PaCa-2		Vero		MIA PaCa-2	
	Gene	Fold change	Gene	Fold change	Gene	Fold change	Gene	Fold change
TGfβ signalling	-	-	*GADD45B*	1.73	*GADD45B*	−1.53	-	-
WNT signalling	*WISP1*	3.13	*WISP1* *AXIN2*	11.12 1.95	*AXIN2*	−1.52	-	-
NFkB signalling	-	-	*STAT1*	1.65			-	-
JAK/STAT signalling	-	-	-	-	*LRG1* *GATA3* *CCND1* *CEBPD*	−4.31 −3.98 −1.63 −1.98	*GATA3*	−2.18
P53 signalling	-	-	-	-	*CDKN1A* *BTG2*	−2.39 −1.83	*CDKN1A*	−1.63
Notch signalling	-	-	-	-	*HES5*	−2.13	-	-
Hedgehog signalling	-	-	-	-	*WNT3A* *BCL2*	−2.87 −1.91	*WNT3A*	1.55
PPAR signalling	-	-	*ACSL4*	1.64	*ACSL4*	−1.68	-	-
Oxidative stress	*TXNRD1*	2.08	*NQO1* *FTH1*	1.68 1.54	-	−	*TXNRD1*	−1.67
Hypoxia	-	-	*LDHA* *ADM* *VEGFA*	1.86 1.66 1.62	*ADM* *SLC2A1*	−2.35 −1.58	*SLC2A1*	−1.54
Housekeeping genes	*ACTB*	2.82	*ACTB*	1.85	*GAPDH*	−1.60	-	-

* Full names of genes are shown in Table S1.

## Data Availability

Data for this study is available upon request.

## Supplementary Materials

The following are available online at https://www.mdpi.com/article/10.3390/life11060462/s1, Detailed PBMC isolation protocol; Figure S1: Line graph showing the effect of BA and PEG–BA IL-6; Figure S2: Effect of BA and PEG–BA on IL-17A, IFN-γ, TNF-α, IL-10, IL-6, IL-4 and IL-2; Figure S3: The 96-plate layout of the human signal transduction RT2 profiler PCR array panel with the position of the genes; Table S1: Full names of dysregulated genes.
