# Supplementary material for: Anti-Cancer and Immunomodulatory Activity of a Polyethylene Glycol-Betulinic Acid Conjugate on Pancreatic Cancer Cells
Source: Life (Basel). 2021 May 21;11(6):462. doi: 10.3390/life11060462 (PMC8223974; doi:10.3390/life11060462)
Supplement: Supplementary file 1 [file life-11-00462-s001.zip › life-1175208-supplementary.pdf]

Supplementary files

A) Isolation of Peripheral Blood Mononuclear Cells

The cells were isolated from whole blood taken from patients and the control group after receiving informed consent and ethics clearance (M170440). After separating the plasma, the blood was diluted in a 1:1 ratio using Roswell Park Memorial Institute (RPMI) medium without foetal bovine serum (Sigma Aldrich, St Louis, MI, USA). The diluted blood was layered onto ficoll at a 2:1 ratio. This was followed by centrifugation at 1734 x *g*, for 30 min, RT with the brakes off. The PBMCs were transferred to a new 50 mL falcon tube and washed (932 x *g*, 10 min RT) with 30 mL of RPMI (Sigma Aldrich). Red blood cells were lysed using ammonium–-chloride–potassium, or ACK (150 mM NH_4Cl, 10 mM $KHCO_3$, 0.1 mM EDTA, pH 7.2) for 5 min before washing with 15 mL of RPMI (277 x *g*, RT, 10 minutes). The cells were resuspended and maintained in RPMI supplemented with 10% FBS, gentamicin, and antibiotic/antimycotic solution (complete RPMI). This was followed by stimulation for 2 h with 2 µg/mL of phytohemagglutinin-protein (PHA–P) also known as lectin from Phaseolus vulgaris (Sigma Aldrich, St Louis, MI, USA) before treatment with compounds.

B) Effect of compounds on IL-6

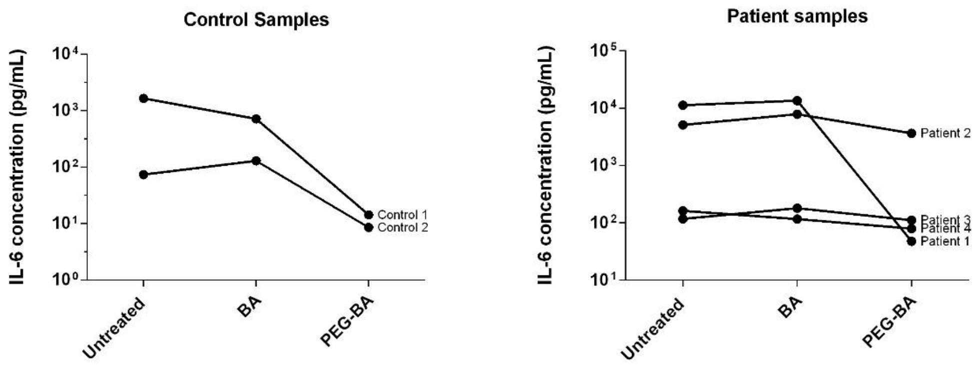

Figure S1: Line graph showing the effect of BA and PEG–BA on IL-6. A notable inhibition of IL-6 by PEG–BA is shown. The *y*-axis scale is Log10, $n = 2$ controls, $n = 4$ patients. Although there was a consistent decrease in IL-6 concentration when patient samples were treated with BA versus PEG–BA, it is evident that the responses for patient 3 and 4 were much reduced. This could be attributed to the varying degrees of inflammation occurring due to differences in cancer severity, i.e., locally advanced versus metastatic.

C) Effect of compounds on Th1Th2Th17 cytokines

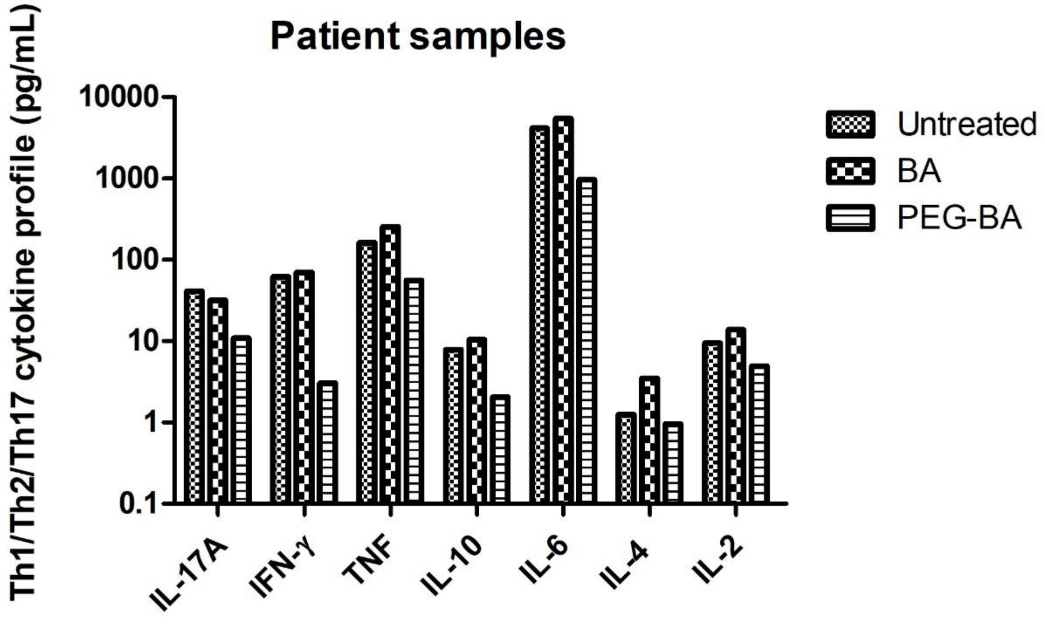

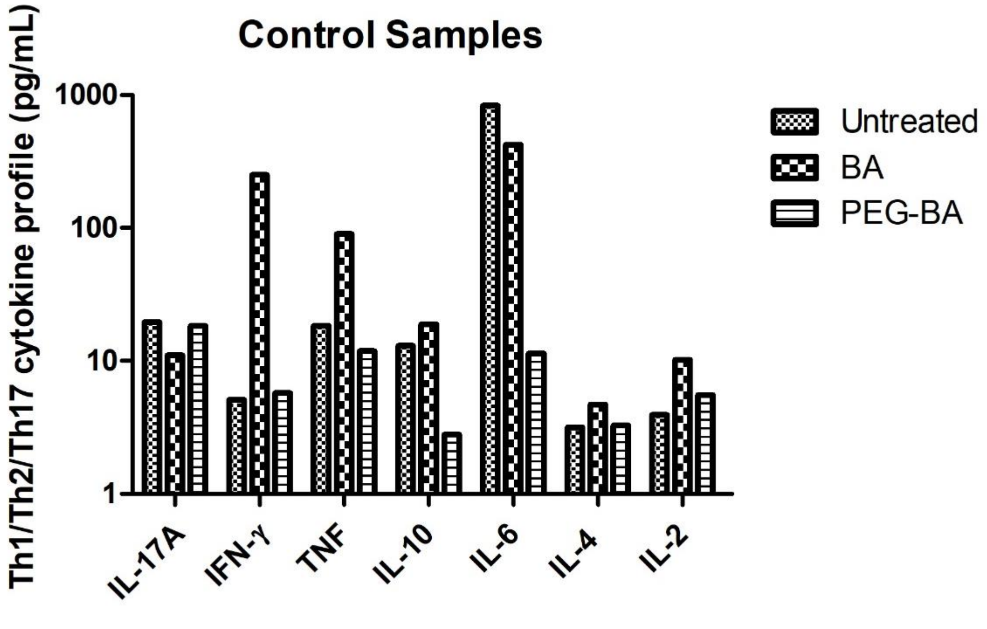

Figure S2: Effect of BA and PEG–BA on IL-17A, IFN-Y, TNF-α, IL-10, IL-6, IL-4 and IL-2. A notable increase in the inhibition of IL-17A, IFN-γ, TNF-α, IL-10 and IL-6 by PEG–BA was shown compared to that induced by BA mainly in the patient samples. The y-axis scale is Log10, n = 2 controls, n = 4 patients.

D) RT2 profiler plate layout

	1	2	3	4	5	6	7	8	9	10	11	12
A	ACSL3	ACSL4	ACSL5	ADM	ARNT	ATF4	AXIN2	BAX	BBC3	BCL2	BCL2A1	BCL2L1
B	BIRC3	BMP2	BMP4	BTG2	CA9	CCL5	CCND1	CCND2	CDKN1A	CDKN1B	CEBPD	CPT2
C	CSF1	DAB2	EGFR	EMP1	EPO	FABP1	FAS	FCER2	FOSL1	FTH1	GADD45A	GADD45B
D	GATA3	GCLC	GCLM	GSR	HERPUD1	HES1	HES5	HEY1	HEY2	HEYL	HMOX1	ICAM1
E	ID1	IFNG	IFRD1	IRF1	JAG1	LDHA	LFNG	LRG1	MCL1	MMP7	MYC	NOTCH1
F	NQO1	OLR1	PCNA	PPARD	PTCH1	RB1	SERPINE1	SLC27A4	SLC2A1	SOCS3	SORBS1	SQSTM1
G	STAT1	TNF	TNFSF10	TXN	TXNRD1	VEGFA	WISP1	WNT1	WNT2B	WNT3A	WNT5A	WNT6
H	ACTB	B2M	GAPDH	HPRT1	RPLP0	HGDC	RTC	RTC	RTC	PPC	PPC	PPC

Figure S3: The 96-plate layout of the human signal transduction RT^2 profiler PCR array panel with the position of the genes.

Table S1: Full names of dysregulated genes

Gene symbol	Gene name
WISP1	WNT1-inducible-signaling pathway protein 1
TXNRD1	Thioredoxin reductase 1
ACTB	Actin Beta
GADD45B	Growth arrest and DNA-damage-inducible, beta
AXIN2	Axin-related protein
STAT1	Signal transducer and activator of transcription 1
ACSL4	Acyl-CoA Synthetase Long Chain Family Member 4
FTH1	Ferritin heavy chain 1
NQO1	NAD(P)H dehydrogenase [quinone] 1
VEGFA	Vascular Endothelial Growth Factor A
ADM	Adrenomedullin
LRG1	Leucine-rich alpha-2-glycoprotein
GATA3	GATA binding protein 3
CCND1	B-cell leukemia/lymphoma 1
CEBPD	CCAAT Enhancer Binding Protein Delta
CDKN1A	Cyclin Dependent Kinase Inhibitor 1A
BTG2	BTG Anti-Proliferation Factor 2
HES5	Hes Family BHLH Transcription Factor 5
WNT3A	Wingless-related integration site 3 A
BCL2	B-cell lymphoma 2
ACSL4	Acyl-CoA Synthetase Long Chain Family Member 4
SLC2A1	Solute carrier family 2 member 1
GAPDH	Glyceraldehyde 3-phosphate dehydrogenase
